# Resistance potentiators: Evolutionary catalysts of antibiotic resistance

**DOI:** 10.1371/journal.pbio.3003852

**Published:** 2026-07-06

**Authors:** R. Craig MacLean, Adam Mulkern, Liam P. Shaw

**Affiliations:** 1 All Souls College, Oxford, United Kingdom‌‌; 2 Department of Biology, University of Oxford, Oxford, United Kingdom; 3 School of Biochemistry and Biomedical Sciences, University of Bristol, Bristol, United Kingdom

## Abstract

Why do even closely-related bacteria differ in their capacity to evolve antibiotic resistance? Drawing on evidence from experimental evolution, pathogen genomics, and molecular microbiology, this Essay argues that the evolution of antibiotic resistance in bacterial genomes is frequently catalyzed by the presence of ‘resistance potentiators’: genes, elements, or pathways that accelerate evolution in a trait-specific manner. Epidemiological evidence suggests that resistance potentiators that modulate phenotypes have been particularly important in successful pathogen lineages. Furthermore, experimental models show that combining antibiotics with inhibitors of resistance potentiators can restrict the evolution of resistance, suggesting that they could be future drug targets or otherwise lead to more evolution-informed antibiotic therapy.

## Abstract 2

How do bacteria evolve resistance to antibiotics? Drawing on evidence from experimental evolution, pathogen genomics, and molecular microbiology, this Essay argues that the evolution of antibiotic resistance in bacterial genomes is frequently catalyzed by the presence of ‘resistance potentiators’: genes, elements, or pathways that accelerate evolution in a trait-specific manner.

## Introduction

The evolution of antibiotic resistance in pathogenic bacteria has caused a global crisis that is undermining human health and prosperity. Although novel antibiotics are crucial to tackle this crisis, we also need to ensure we are making the best use of the antibiotics we already have. We often know the evolutionary mechanisms that lead to antibiotic resistance [1–3], but to inform treatment strategies, it would be useful to accurately predict which bacterial strains are most at risk of adapting to antibiotics. However, recent studies have shown that even closely-related pathogenic bacteria differ in their abilities to evolve specific resistances [4–7]. This variability implies that there are genetic factors that make bacteria more likely to evolve a particular resistance phenotype. What are these factors?

The evolution of antibiotic resistance can occur both by novel mutations and by the acquisition of new genes by horizontal gene transfer. We would therefore expect that any resistance phenotype will evolve more rapidly in bacterial genomes that have a high mutation rate (Box 1) or are prone to accumulating new mobile genetic elements (Box 1). In both cases, the evolution of antibiotic resistance is merely a byproduct of enhanced genomic plasticity (Box 1), which is a proxy for general evolvability for any trait. In this Essay, we argue that to understand the evolution of antibiotic resistance we need to go beyond the concept of general evolvability. Instead, we should search for genetic factors that influence the evolution of antibiotic resistance in a trait-specific manner. Recent studies have identified numerous factors that can pre-dispose bacteria to evolve antibiotic resistance, but a consistent terminology is lacking: specific instances have been described as “pre-resistance mutations” [4], “metabolic mediators” [5], or as an “evolutionary gateway” [6]. Here, we present a conceptual framework that unites them.

**Box 1. Glossary.**

**Mutation rate**

The rate at which mutations occur during cell replication.

**Mobile genetic elements**

Genetic elements that are capable of horizontal transmission between bacterial cells, such as plasmids and temperate phage.

**Genomic plasticity**

Plastic genomes undergo a high rate of change due to a high mutation rate and/or rapid turnover in mobile genetic elements.

**Hypermutator**

Bacterial variant with a constitutively elevated mutation rate due to mutations in genes involved in DNA replication and repair, such as mutants with a defective methyl-directed mismatch repair pathway.

**Intrinsic resistance genes**

Chromosomally encoded and conserved ‘core’ genes in bacterial genomes that provide a baseline of protection to antibiotics. Intrinsic resistance genes often protect against multiple stressors (i.e., efflux pumps), but some have specialized roles in protecting cells against antibiotics (i.e., core β-lactamase enzymes).

In general, the evolution of a new trait is dependent on preexisting genetic factors. For example, in a seminal series of papers [8], Lenski and colleagues demonstrated that adaptation of *Escherichia coli* to a glucose-based culture medium led to the evolution of lineages that were more likely to evolve aerobic citrate metabolism—an extremely surprising evolutionary innovation. The evolution of this novel trait was not accelerated by generalized hypermutability. Instead, it was historically contingent on the presence of a subset of mutations, which the authors dubbed “potentiating” mutations, that accumulated during adaptation to glucose [7]. By analogy, the evolution of resistance is also historically contingent. Broadening the concept of Lenski and colleagues, it can be accelerated by potentiating genes, elements, or pathways that are trait-specific. The presence of these factors can make some bacteria more evolvable when it comes to antibiotic resistance than others. We define these diverse factors as **resistance potentiators**: the increased evolvability they confer is specific to antibiotic resistance rather than merely a reflection of high genomic plasticity.

In this Essay, we discuss resistance potentiators, drawing on evidence from experimental evolution, pathogen genomics, and molecular microbiology. The effects of resistance potentiators may be subtle, but they can now be identified in genomes using a range of computational and experimental approaches (Box 2). Broadly, potentiators accelerate the evolution of antibiotic resistance by either increasing the rate at which resistance is acquired or by epistatically modifying the phenotypic effects of resistance determinants (i.e., antibiotic resistance level, fitness costs). One of our central arguments is that resistance potentiators are important drivers of antibiotic resistance in clinical pathogens. A better understanding of their effects may therefore help our understanding of antibiotic resistance evolution, and hopefully offer new ways to combat it.

**Box 2. Methods for identifying resistance potentiators.**

Genomes carrying resistance potentiators are expected to be associated with antibiotic resistance genes. One way to test for genes that act as resistance potentiators is therefore to use computational methods, such as genome-wide association studies, to search for single nucleotide polymorphisms or genes that show statistical association with antibiotic resistance genes [5,9–11]. This approach is intuitive and potentially very powerful, but one important limitation of using genome comparisons to identify resistance potentiators is that the antibiotic pressure faced by pathogen lineages is variable. For example, lineages that are associated with geographic areas of high antibiotic use [4], human carriage [12], or host immune evasion [4,13] are expected to have higher antibiotic resistance simply because they are exposed to higher levels of antibiotics. Variation in antibiotic exposure is widespread, and it is challenging to account for this potentially confounding variable using computational approaches alone (see [14] for an excellent example). Acquiring antibiotic resistance genes carries fitness costs [15,16], generating selection for compensatory mutations that alleviate the cost of resistance [17]. Compensatory mutations that are acquired following the acquisition of antibiotic resistance genes are also expected to show associations with antibiotic resistance genes [18–21], generating a risk of confounding potentiators with compensatory genes. In principle, it should be possible to circumvent these problems by using phylogenetic methods to reconstruct the chronology of acquisition of mutations associated with antibiotic resistance. However, in many cases antibiotic resistance evolves rapidly enough relative to the time scale in which pathogen populations are sampled that this approach is not feasible [14]. Given these constraints, candidate genes that are identified as putative resistance potentiators by computational methods should be functionally assessed to test their impact on the rate of acquisition of antibiotic resistance genes or the phenotypes (e.g., fitness costs, resistance levels) associated with antibiotic resistance genes [5,6,22].

Experimental evolution under controlled lab conditions can also be used to identify resistance potentiators. This approach makes it possible to account for potentially confounding variables, such as differences in antibiotic exposure and population size between bacterial populations, but this approach relies on using simplified model systems that do not fully recapitulate the conditions that bacteria encounter in real-world settings. In the simplest case, experimental evolution has been used to compare the evolvability of wild-type bacteria compared to mutants lacking candidate resistance potentiators [23,24]. High-throughput evolution experiments using systematic mutant collections can also be used to identify genes acting as novel resistance potentiators. For example, systematic genomic screening found that efflux pumps and genes involved in protein folding potentiate the evolution of tetracycline resistance in *Escherichia coli* [25]. Alternatively, experimental evolution can be used to compare the evolvability of panels of clinical and environmental isolates [22–24,26]. The advantage of this approach is that it directly tests naturally occurring genetic variation and can potentially capture subtle variation in expression levels and protein structure that are lacking from knock-out mutant libraries. The main downside of this approach is that it is much more challenging to infer the mechanistic basis of potentiation given that isolates differ from each other in genome composition (i.e., gene presence/absence) and nucleotide variation. In these studies, putative resistance potentiators have been identified through analyses of antibiotic resistance evolution mechanisms or transcriptomic signatures associated with high-evolvability strains [23,24].‌‌

## Stress response pathways

The simplest mechanism for bacteria to evolve antibiotic resistance is by acquiring mutations in existing genes, which is the dominant mechanism of resistance for some classes of antibiotics and pathogens [27]. In these cases, it is obvious that increasing the overall mutation rate will accelerate the evolution of antibiotic resistance. For example, mutations in the DNA replication and repair pathways lead to a hypermutator (Box 1) phenotype that is associated with a constitutive increase in the mutation rate. Hypermutators accelerate the evolution of antibiotic resistance [28–35], but they are not resistance potentiators by our definition because the increase in evolvability they generate is not specific to antibiotic resistance. For example, hypermutators also accelerate adaptation to phage [36], lab culture medium [37], and the mouse gut [38].

**Fig 1:**
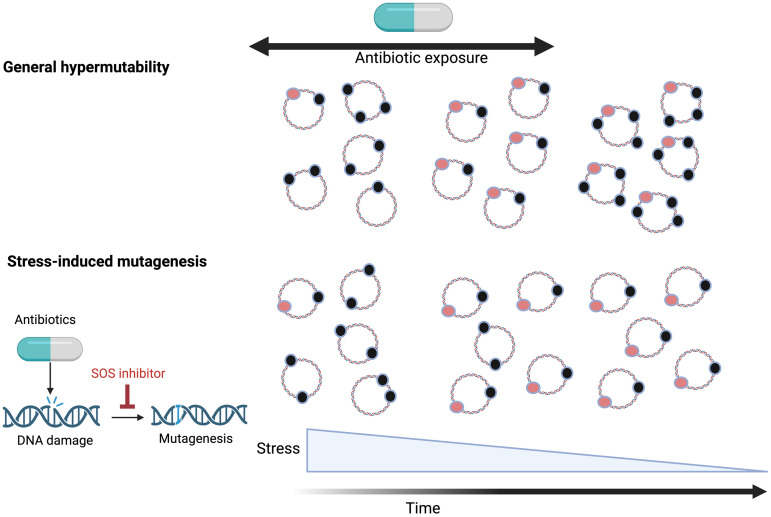
Mutagenesis and the evolution of antibiotic resistance. A schematic of mutagenesis mechanisms as drivers of antibiotic resistance evolution. Mutations are represented as filled circles in bacterial chromosomes from hypothetical populations, with antibiotic resistance mutations shown in red and weakly deleterious mutations shown in black. Hypermutator populations have a constitutively elevated mutation rate. Hypermutation drives the rapid evolution of antibiotic resistance, but ultimately results in the accumulation of a large load of deleterious mutations. In populations with stress-induced mutagenesis, exposure to antibiotics generates DNA damage that triggers the expression of inducible error-prone DNA polymerases through regulatory pathways such as the SOS pathway, resulting in a burst of mutagenesis. Adaptation to antibiotics results in decreased stress and a suppression of stress-induced mutagenesis, limiting the accumulation of deleterious mutations. This potentiator mechanism can be inhibited using drugs that block the SOS pathway, shown with a red inhibitor arrow. Figure created in BioRender. https://BioRender.com/wg4q4nb.

However, another way of increasing the mutation rate is via stress-induced mutagenesis. Exposure to antibiotics damages the cell, triggering the expression of stress response pathways that are associated with inducible mutagenesis, stemming from the expression of error-prone DNA polymerases or the suppression of DNA repair mechanisms [39,40]. As expected, stress-induced mutagenesis accelerates the evolution of antibiotic resistance, and inhibiting it has been proposed as a way of combating antibiotic resistance [41,42]. Given that mutagenic polymerases generate mutations at random across the genome, stress-induced mutagenesis could be argued to be a case of general hypermutability. However, a key difference is that stress-induced mutagenesis is transient: adaptation to antibiotics rapidly leads to the suppression of stress response pathway expression [43], whereas constitutive mutagenesis associated with hypermutators ensures that bacterial lineages continually accumulate a genetic load of unselected mutations [44,45] (Fig 1). Perhaps for this reason, evidence suggests that inducible mutators contribute more to natural variation than constitutive mutators in wild bacterial populations [39]. Theoretical modeling shows that stress-induced mutagenesis can increase the rate of adaptation without reducing the population’s mean fitness, supporting the view of it as a resistance potentiator [46].

Stress response pathways also induce the activity of mobile integrons, which consist of an integrase followed by an array of antibiotic resistance cassettes [47,48]. Integrase activity generates combinatorial variation in cassette composition and expression levels through the insertion and deletion of cassettes that are expressed from a common promoter [49]. This variation accelerates the evolution of antibiotic resistance by fine-tuning cassette expression and eliminating redundant cassettes that impose a fitness cost [50].

The antibiotic-inducible and transient increase in genetic variation provided by stress-induced mutagenesis and integrons accelerates the evolution of antibiotic resistance in a trait-specific manner. Therefore, we view them as a form of resistance potentiator (albeit a debatable one, because they accelerate adaptation to any stressor that induces stress responses, and not just antibiotics). We now turn to more clear-cut examples.

## Increased expression of intrinsic resistance genes

Bacterial genomes contain intrinsic resistance genes (Box 1) that provide cells with a baseline of protection to antibiotics [51]. Increasing the expression of intrinsic resistance genes, such as those encoding efflux pumps and antibiotic-degrading enzymes [27], provides a simple mechanism to evolve increased antibiotic resistance. In this model of evolution, genes that allow the expression levels of intrinsic resistance genes to be rapidly increased act as resistance potentiators. For example, the evolution of antibiotic resistance by increased efflux pump activity is often driven by mutations that inactivate transcriptional repressors of efflux pump operons [52,53]. Here, efflux pump repressors behave as resistance potentiators. An alternative mechanism to increase the expression of intrinsic resistance genes is gene amplification, which can result from nonhomologous recombination between repeated sequences such as ribosomal RNA operons or insertion sequence elements. For example, *Salmonella typhimurium* evolves resistance to cephalosporin antibiotics by the amplification of a region of the chromosome carrying a ß-lactamase with a weak ability to degrade cephalosporins [54]. Gene amplification events can occur at a much higher frequency than mutations [55,56] and repeated sequences that allow for gene amplification act as resistance potentiators by generating extensive variation in the expression level of antibiotic resistance genes [57,23]. In some ways, this is analogous to the variability in antibiotic resistance gene expression levels generated by integrons. However, gene amplification typically involves regions of the chromosome that are much larger than the antibiotic resistance genes themselves [54,58], suggesting that gene amplification is a costly mechanism for increasing expression relative to integron cassette re-arrangement.

Gene amplification is common in lab experiments [55,59] and has been detected in clinical settings [60–62]. In some cases, gene amplifications are inherently unstable and can only be maintained under continuous antibiotic pressure [57], suggesting that some amplification events are effectively lost during the culturing of bacterial isolates from clinical samples prior to phenotypic and genomic analysis, biasing clinical studies against detecting gene amplification. However, in other cases, gene amplification can be stably maintained in the lab in the absence of antibiotic pressures [58], suggesting that high *in vivo* fitness costs may eliminate them in clinical settings.

## Plasmids

Many of the most important antibiotic resistance phenotypes have evolved through the acquisition of plasmids [63] that provide bacteria with effective resistance mechanisms at a low fitness cost compared to mutations [15]. Models of plasmid transfer have classically been based on the idea that plasmid transfer is firmly controlled by plasmid-carrying donor cells [64]. However, it has become clear that plasmid acquisition is also regulated at multiple layers in recipient cells.

The transfer of plasmids depends on binding between plasmid-encoded transfer proteins on donor cells and complementary receptors in recipient cells, suggesting that genes encoding receptor proteins act as resistance potentiators by determining the acquisition of specific plasmids associated with antibiotic resistance genes [65–67]. Plasmids can only establish themselves in a new host if they are not targeted by xenogenic defense systems that act as a second barrier to plasmid acquisition [22,68–72]. Anti-defense systems that suppress these barriers, therefore, also have the potential to act as resistance potentiators by accelerating plasmid acquisition. Plasmid acquisition is usually associated with fitness costs, forming a final barrier to the formation of stable bacteria–plasmid associations. Fitness costs associated with plasmid acquisition are complex [73], but one emerging theme is that regulatory cross-talk between plasmids and the chromosome underpins the formation of successful associations, highlighting the importance of genes encoding chromosomal regulatory proteins as potentiators for plasmid acquisition, and thus as resistance potentiators [74–78].

The genes that either permit or constrain plasmid acquisition at each of these levels are specific to individual plasmids. In other words, the available evidence suggests that bacteria lack ‘master regulators’ for plasmid acquisition that would be analogous to hypermutators. This idea is further supported by the observation that plasmids carrying antibiotic resistance genes are often associated with specific bacterial lineages, suggesting that some bacterial chromosomes are more likely to acquire specific plasmids than others [79–81]. For example, similar mutational profiles in gene regulatory regions between clades of *E. coli* ST131 may explain their acquisition of similar antibiotic resistance plasmids [82]. Chromosomal factors influencing plasmid acquisition can thus behave as resistance potentiators, and could explain the differential success of epidemic lineages.

## Amplified resistance phenotypes

**Fig 2:**
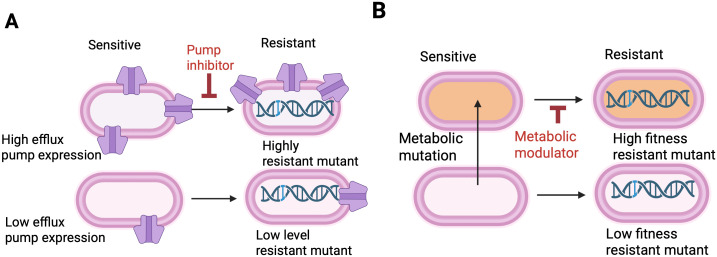
Epistatic potentiators of antibiotic resistance evolution. **A**. High expression of efflux pumps accelerates the evolution of antibiotic resistance by increasing the phenotypic impact of mutations that alter antibiotic targets. This potentiator pathway can be blocked using efflux pump inhibitors. **B**. Mutations in metabolic genes potentiate the evolution of antibiotic resistance by establishing robust metabolic networks that minimize the fitness costs associated with antibiotic resistance mutations. This pathway of potentiation could conceivably be blocked by metabolic modulators. Figure created in BioRender. https://BioRender.com/jt7dem0.

Another possible mechanism to accelerate the evolution of antibiotic resistance is by increasing the phenotypic impact of ‘canonical’ antibiotic resistance genes as a result of positive epistasis between potentiator genes and resistance genes (Box 3). For example, strains of *Staphylococcus aureus* with high NorA efflux pump activity evolve resistance to ciprofloxacin by facilitating the acquisition of resistance mutations in DNA gyrase and topoisomerase [23]. Efflux pumps often act as important antibiotic resistance genes [27], but in this case efflux pump activity does not provide increased resistance to ciprofloxacin per se. Rather, NorA activity interacts epistatically with mutations that alter the targets of ciprofloxacin (i.e., DNA gyrase and topoisomerase) to increase ciprofloxacin resistance, accelerating the evolution of resistance (Fig 2A). Large numbers of genes in bacterial genomes are associated with subtle effects on antibiotic resistance [83,84], and we speculate that many of these genes accelerate adaptation to antibiotics by epistatically modifying the phenotypic effects of canonical antibiotic resistance genes.

**Box 3. Epistasis and antibiotic resistance.**

Early breeding experiments with animals and plants demonstrated that phenotypes associated with alleles at one locus can be masked by the alleles present at other loci, giving rise to the concept of epistasis [85]. More generally, epistasis occurs when alleles at different loci interact to determine phenotypes, including fitness. Epistasis for quantitative traits between two loci (A and B) in a haploid organism can be represented as w_AB_ = (w_aB_*w_Ab_) + e_AB,_ where w_aB_, w_Ab_, and w_AB_ are the phenotypic values for the single mutants and double mutant relative to the wild-type, respectively, and e_AB_ is the epistatic interaction term [86,87]. This model of epistasis can be applied to any quantitative trait (i.e., fitness, growth rate, antibiotic minimum inhibitory concentration) that can be measured relative to a wild-type reference. Positive epistasis (i.e., e_AB_ > 0) occurs when the trait value of the double mutant is greater than expected, and negative epistasis (i.e., e_AB_ < 0) occurs when the trait value of the double mutant is smaller than expected. In extreme cases, epistasis results in mutations changing the sign (i.e., + or −) of their phenotypic effect in different genetic backgrounds (i.e., sign epistasis) [88]. As a hypothetical example, consider a scenario where two mutations individually increase antibiotic resistance compared to a wild-type reference strain, but where the double mutant has reduced resistance compared to the wild-type.

In other cases, genes that are not linked to antibiotic resistance in any obvious way act as potentiators through epistatic interactions with resistance genes. For example, chromosomal genes with diverse cellular functions are required for the horizontally acquired *mecA* gene to generate a methicillin-resistant phenotype, and these genes are therefore epistatic potentiators for the evolution of methicillin-resistant *S. aureus* (MRSA) [89]. In some cases, resistance potentiators can even be associated with antibiotic sensitivity. For example, the evolution of high-level colistin resistance in pathogenic *E. coli* was driven by the acquisition of plasmids carrying the *mcr-1* colistin resistance gene in strains carrying polymorphisms in *lpxC*. Mutations in *lpxC* are associated with decreased colistin resistance in the absence of *mcr-1*, providing a clear-cut example of sign epistasis (Box 3) between resistance genes and potentiators [90].

Antibiotic resistance is often treated as a binary trait (i.e., resistant or sensitive) because of its clinical relevance, but the evolution of resistance to the high levels of antibiotics encountered during a therapeutic course is often driven by the stepwise acquisition of multiple resistance determinants. One of the most general findings of experimental evolution has been that the evolution of fitness (or phenotypic traits correlated with fitness) slows over time, and this deceleration is driven by negative epistasis between beneficial mutations [91–93]. Accordingly, we would expect that the early mutations that spread under antibiotic pressure will be associated with large phenotypic effects, and that secondary mutations would be associated with comparatively small changes.

Although some experimental studies have supported this diminishing returns model of adaptation to antibiotics [94], others have shown that the initial adaptive changes under antibiotic pressure lead to small changes, but act as an important evolutionary stepping stone for subsequent adaptations. For example, the evolution of MRSA is driven by the acquisition of *mecA* that provides low level resistance, followed by the acquisition of secondary resistance mutations that enhance or refine the resistance provided by *mecA* [95]. Similarly, the evolution of resistance to antibiotics that inhibit cell wall synthesis in *Streptococcus pneumoniae* is caused by the acquisition of resistance mutations in *Pde1*, followed by the acquisition of mutations in penicillin-binding proteins that confer higher levels of resistance [6]. Finally, the evolution of high-level colistin resistance in *Pseudomonas aeruginosa* is potentiated by the initial acquisition of mutations in regulatory genes that provide low-level colistin resistance [96].

In all of these cases, the evolutionary trajectory to high-level resistance is characterized by the initial acquisition of mutations in a gene that acts first as a resistance gene by conferring a low level of protection to the antibiotic, and then subsequently as a resistance potentiator (because the subsequent mechanisms that confer high levels of resistance are contingent on the presence of the mutated form). In these cases, positive epistasis occurs between the primary resistance determinants that provide the initial baseline of protection against antibiotics and the secondary resistance determinants associated with high-level resistance.

## Phenotypic buffering and fitness costs

Acquiring antibiotic resistance usually incurs a fitness cost, expressed in terms of reduced competitive ability in the absence of antibiotics [15–17]. Fitness costs can lead to the rapid loss of resistance in bacterial populations when antibiotic pressure is reduced [17,97–99], and it has been argued that fitness costs are the key determinant of the spread and stabilization of antibiotic resistance in pathogen populations [1,17,100–104].

Fitness costs of acquiring antibiotic resistance vary across genetic backgrounds [26,105–107], implying that some bacterial strains have genomes that are inherently robust to the cellular perturbations associated with acquiring antibiotic resistance genes because they carry genes that epistatically modify the fitness costs associated with the resistance genes. For example, in *Neisseria gonorrhoeae*, the CanB^19G^ polymorphism accelerates the evolution of ciprofloxacin resistance by reducing the fitness costs associated with acquiring mutations in *gyrA* [5]. This positive epistasis for fitness arises because CanB^19G^ alters metabolism in a way that makes the cell robust to deleterious consequence of DNA gyrase mutations. Altered metabolism also has a role in potentiating the evolution of fluoroquinolone resistance in *E. coli* [14], suggesting that this is indicative of a broader link between metabolic genes and resistance determinants (Fig 2B). Worryingly, the combined fitness cost of carrying multiple antibiotic resistance mutations is often less than would be expected based on the fitness effects of the individual mutations, revealing widespread positive epistasis for fitness between resistance determinants [86,108–110]. As a consequence, adaptation to one antibiotic can potentiate the evolution of resistance to subsequent antibiotics, facilitating the emergence of multi-drug resistance.

## Combatting resistance

We have introduced the concept of resistance potentiators and argued that it provides a useful framework for thinking about the evolution of antibiotic resistance. But how could developing a better understanding of potentiators practically help to combat antibiotic resistance? One possibility is that understanding routes to resistance evolution can make it easier for clinicians to choose antibiotic therapies with a lower risk of resistance [35,111]. At the moment, these take the form of general rules: for example, rifampicin is not recommended as a single agent to treat *S. aureus* because resistance is known to arise via a single mutation. But as whole–genome sequencing becomes more common and our understanding of evolution improves, routine diagnostic panels could include resistance potentiators as well as predicted antibiotic resistance phenotypes, allowing tailored treatment for individual patients, such as combination therapy when a known potentiator is present.

**Fig 3:**
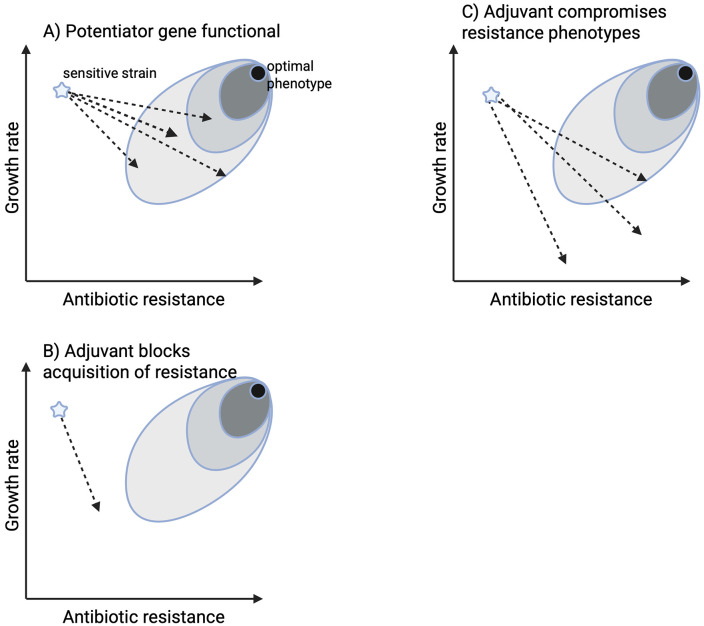
Evolutionary trajectories to resistance. Hypothetical evolutionary trajectories followed by a sensitive strain (star) under antibiotic pressure. Each arrow shows a hypothetical beneficial mutation and the shaded regions show phenotypes with progressively higher fitness. In panel **A**, the potentiators in the sensitive strain are intact. Panels **B** and **C** show hypothetical evolutionary trajectories when adjuvants block potentiators that accelerate the acquisition of resistance (B) or that amplify antibiotic resistance phenotypes (C). Figure created in BioRender. https://BioRender.com/9ow1603.

However, resistance potentiators also offer more direct strategies to prevent the evolution of antibiotic resistance. One possibility is to use adjuvants that inhibit known potentiators (Figs 1 and 3). For example, antibiotics can be combined with compounds that suppress the expression of stress response pathways [42,112]. This is a particularly promising strategy for preventing the evolution of resistance in acute infections where the pathogen population has undergone a recent population bottleneck, ensuring that little-to-no genetic diversity is present in the pathogen population at the onset of treatment. For plasmids, conjugative transfer can be suppressed using chemical inhibitors [113] or by treatment with lytic phage that use the conjugative pilus as a receptor [114]. Neither of these strategies actually targets potentiator mechanisms, although they achieve the same goal of inhibiting transfer. Direct targeting of potentiators, such as receptor protein expression in recipient cells, could represent another line of attack.

Arguably, the first combination therapy that was directly informed by knowledge of resistance mechanisms was the use of ß-lactamase inhibitors in combination with ß-lactam antibiotics. An extension of this idea is inhibiting potentiators that epistatically modify the effects of resistance genes rather than targeting the resistance genes themselves (Fig 3). Some efflux pumps and ß-lactamases only provide low-level protection against antibiotics, meaning they act primarily as potentiators that accelerate resistance evolution and are not as important resistance mechanisms themselves. This distinction is critical because selection for resistance to an inhibitor of a potentiator gene in these cases will be weaker than it would be if the inhibitor were being used to directly target a high-level resistance gene. This small selective benefit of adapting to overcome inhibitors of potentiators makes it very challenging for bacterial populations to adapt to combinations of antibiotics and potentiator inhibitors. For example, combining antibiotics with adjuvants that inhibit ‘weak’ efflux pumps and ß-lactamases that act as potentiators can substantially reduce the rate of evolution of antibiotic resistance under lab conditions [23,24,53].

## Conclusions and future directions

Antibiotic resistance does not evolve at equal rates, even among closely-related bacteria. These differences are driven by genetic factors that can accelerate the rate of acquisition of antibiotic resistance genes, amplify the protection they confer, or provide robustness to their deleterious side-effects. We have argued that these diverse factors can be conceptually grouped together as resistance potentiators. In some cases, individual genes act as resistance potentiators, such as *canB, lpxC*, or *norA.* In others, potentiation comes from multiple genes acting in concert, such as a stress response pathway that induces mutagenesis or integron activation in response to antibiotic therapy.

Which potentiator mechanisms are the most important in clinical settings? Strikingly, successful pathogen lineages tend to be associated with resistance potentiators that epistatically modify the phenotypic effects of antibiotic resistance genes [4–6,14,89,90]. This trend supports the idea that resistance is limited by the realized phenotypic effects of antibiotic resistance genes in a given genetic background, rather than the rate at which resistance is acquired [1,115]. The caveat to this argument is that it is based on epidemically successful lineages; it remains possible that high rates of resistance acquisition are much more important at the scale of individual infection [13].

One important challenge for future research will be to understand the mechanisms driving the spread of potentiator genes in pathogen populations. Many genes that act as resistance potentiators themselves provide low levels of antibiotic resistance, suggesting that they are present in pathogen lineages with a history of exposure to low levels of antibiotics, either naturally occurring (i.e. produced by microbes) or anthropogenic. In some cases, these are mechanisms that provide protection against a wider range of stressors, such as efflux pumps, and they may also reflect past selection for general stress tolerance.

It is more challenging to explain the existence of potentiator mechanisms that are not associated with any direct benefits in the presence of antibiotics, such as mechanisms that accelerate the acquisition of resistance or provide robustness to the perturbations associated with it. One possible explanation is that these are ‘accidental’ potentiator mechanisms that have accumulated in pathogen populations for some alternative function, or simply because of genetic drift. For example, pathogenic lineages of *E. coli* may have accumulated *lpxC* mutations that potentiate the evolution of colistin resistance because of recurrent population bottlenecks rather than adaptation to a pathogenic lifestyle. Similarly, there is no evidence that the CanB^19G^ polymorphism provides robustness to perturbations other than those associated with ciprofloxacin resistance, suggesting that it may also be an ‘accidental’ potentiator gene.

Alternatively, it is possible that these potentiators have spread as a result of second-order selection due to linkage between potentiators and beneficial variants they generate [44,116,117]. For example, in integrons, the integrase recombines cassettes that are located immediately downstream, generating a linkage between recombinogenic integrases and the optimized arrays of cassettes they can create. It is certainly conceivable that selection has favored the evolution of ‘evolvable’ regulatory architectures for antibiotic resistance genes, such as those that are inherently prone to mutations or that contain repeated sequence motifs, to provide a template for gene amplification of flanking genes. But while the argument that resistance potentiators exist because of selection for evolvability may be attractive, strong evidence is lacking. We suggest that the default assumption should be that potentiators are ‘accidental’ features of bacterial genomes, as opposed to switches that spread due to the enhanced evolvability that they confer. A challenge for the field moving forward will be to rigorously test these alternative hypotheses for their evolutionary origins.

In this Essay, we have deliberately defined resistance potentiators broadly. While some readers may take issue with this grouping together of diverse mechanisms under a single term, we feel this broad concept provides a helpful lens through which to understand antibiotic resistance evolution. Indeed, the concept of a ‘resistance gene’ is also extremely broad! One potential difficulty with our concept is that many antibiotic resistance potentiators may accelerate evolutionary adaptation to stress in general, such as stress-induced mutagenesis or mutation-prone regulatory circuits for efflux pumps that protect against a wide range of toxins. These potentiator mechanisms are likely to lie somewhere between two extremes: at one end, an idealized potentiator that has no functional effect other than to accelerate the evolution of antibiotic resistance, and at the other, an idealized mechanism of genomic plasticity that accelerates the creation of genetic variation without any pleiotropic side-effects. We think that the potentiator mechanisms discussed in this Essay are more similar to the former than the latter, but an important goal for future research will be to test the impact of antibiotic resistance potentiators on the evolution of other traits.

Fortunately, it is not necessary to agree on a precise definition to use the approach: adjuvants targeting specific resistance potentiators have demonstrated the potential to prevent the evolution of antibiotic resistance *in vitro*. We think these results should be treated with cautious optimism, due to the profound differences that exist between infections and lab culture systems, but it does suggest that identifying novel resistance potentiators could provide future drug targets. Previous attempts to target stress-induced mutagenesis, such as targeting the SOS response [42], have not proved successful in clinical settings. But in principle, the more trait-specific a resistance potentiator is to a given phenotype, the easier it should be to disrupt. Although antibiotic–potentiator adjuvant combinations can be effective, bacterial pathogens will ultimately be able to evolve bypass mechanisms that allow them to circumvent adjuvants, either through adjuvant resistance or by following alternative evolutionary trajectories to resistance. Inhibiting potentiators is likely to delay, rather than prevent, the evolution of resistance.

Antibiotic resistance is inevitable, but ever since the introduction of antibiotics, improvements in our understanding of evolution have informed treatments that reduce its emergence—from combination therapy to ß-lactamase inhibitors. Future work that expands our understanding of resistance potentiator mechanisms, and establishes whether they can be efficiently suppressed during real infections, will become part of this ongoing history.
